# Understanding spiritual well-being in patients with systemic sclerosis and its effect on the illness: a scoping review

**DOI:** 10.1007/s10067-025-07689-1

**Published:** 2025-09-17

**Authors:** Stella Gkountinaki, Foula Protopapa, Amit Syal, Andreas Chatzittofis, Konstantinos Parperis, Chris T. Derk

**Affiliations:** 1 University of Cyprus Medical School, Nicosia, Cyprus; 2 European University of Cyprus School of Medicine, Nicosia, Cyprus; 3 Sidney Kimmel Medical College, Thomas Jefferson University, Philadelphia, USA; 4 Department of Psychiatry, University of Cyprus, Nicosia, Cyprus; 5 Division of Rheumatology, University of Cyprus, Nicosia, Cyprus; 6 Division of Rheumatology, University of Pennsylvania, Philadelphia, USA

**Keywords:** Coping skills, Patient centered care, Personal satisfaction, Psychological resilience, Quality of life, Scleroderma, Spirituality, Systemic sclerosis

## Abstract

**Background:**

Spirituality has become an increasingly important domain of care in chronic diseases, yet little is known about its impact in systemic sclerosis (SSc), a rare autoimmune condition associated with high psychosocial burden. Understanding the spiritual well-being of these patients may offer important insights into coping, quality of life, and patient-centered interventions.

**Methods:**

This scoping review followed PRISMA guidelines and included a comprehensive search of five databases for studies addressing spirituality in patients with SSc. We extracted demographic and methodological details from each study and assessed quality using the Joanna Briggs Institute (JBI) criteria.

**Results:**

Ten studies met inclusion criteria. Spirituality was frequently associated with improved psychological outcomes, including reduced depression and anxiety, increased life satisfaction, and greater resilience. Instruments used to assess spirituality included FACIT-Sp, MI-RSWB, and STS, among others. However, heterogeneity in methodology and limited focus on SSc-specific populations remain notable.

**Conclusion:**

Spiritual well-being plays a significant role in the lived experience of patients with SSc. Future studies should standardize measurement tools and examine culturally sensitive, spirituality-integrated interventions. Incorporating spiritual assessment into rheumatologic care may enhance patient outcomes.

**Table:** 

**Key Points** •*Spiritual well-being is increasingly recognized as a critical domain of health in patients with chronic illnesses, including systemic sclerosis.* •*Spirituality is a relevant and influential factor in the lived experience of SSc. It affects not only how patients understand and cope with their illness but also how they engage with healthcare systems.* •*Spirituality was frequently associated with improved psychological outcomes, including reduced depression and anxiety, increased life satisfaction, and greater resilience.*

**Supplementary Information:**

The online version contains supplementary material available at 10.1007/s10067-025-07689-1.

## Introduction

In recent decades, there has been growing recognition that spiritual well-being can influence health outcomes, particularly in the context of chronic or life-altering illnesses and spirituality has emerged as a meaningful component of patient-centered care across many different medical specialties [1].

Studies have shown that spiritual distress may exacerbate suffering, while spiritual resources can promote resilience, coping, and improved quality of life [2, 3]. As a result, professional organizations, including the World Health Organization (WHO) and the Joint Commission, have called for greater integration of spiritual assessment and support into routine clinical care [4, 5].

Within the field of rheumatology, research exploring spirituality is less well established but steadily expanding. Autoimmune and connective tissue diseases often impose significant physical limitations and psychological burdens, making spiritual coping particularly relevant. Conditions such as lupus, rheumatoid arthritis, and vasculitis have been associated with altered self-identity, uncertainty about disease progression, and increased emotional distress — all areas where spirituality may play a role in meaning-making and adaptation. A small but growing body of literature has begun to examine how spiritual beliefs, practices, and support networks may shape patients’ illness experiences, treatment adherence, and psychological well-being [6–8].

Systemic sclerosis (SSc), or scleroderma, represents a uniquely complex and often debilitating rheumatic disease characterized by progressive fibrosis, vasculopathy, and multi-organ involvement [9]. Patients with SSc frequently contend with visible disfigurement, pain, fatigue, and uncertainty regarding prognosis — factors that contribute to high rates of anxiety, depression, and social isolation [10].

Despite these challenges, few empirical studies have directly explored the role of spirituality in the lived experience of individuals with SSc. Understanding how patients draw on spiritual frameworks in the context of SSc may offer valuable insights for holistic, patient-centered care.

Thus, this scoping review seeks to explore how spirituality influences the experience of illness and the clinical response to it, in patients living with SSc, through the synthesis of the existing literature and identification of prevailing themes, knowledge gaps, and implications for patient-centered care.

In this review, we define spirituality as the way individuals seek and express meaning, purpose, and connection, which may or may not be linked to organized religion. Religiosity refers to the degree of engagement with religious beliefs, rituals, or institutions. Existential well-being denotes a sense of meaning and purpose in life, while self-transcendence describes expansion of self-boundaries to connect with others, nature, or a higher power. Throughout the manuscript, we use “spiritual well-being” as the overarching construct, while recognizing these related but distinct dimensions.

## Patients and methods

### Search strategy

**Fig. 1:**
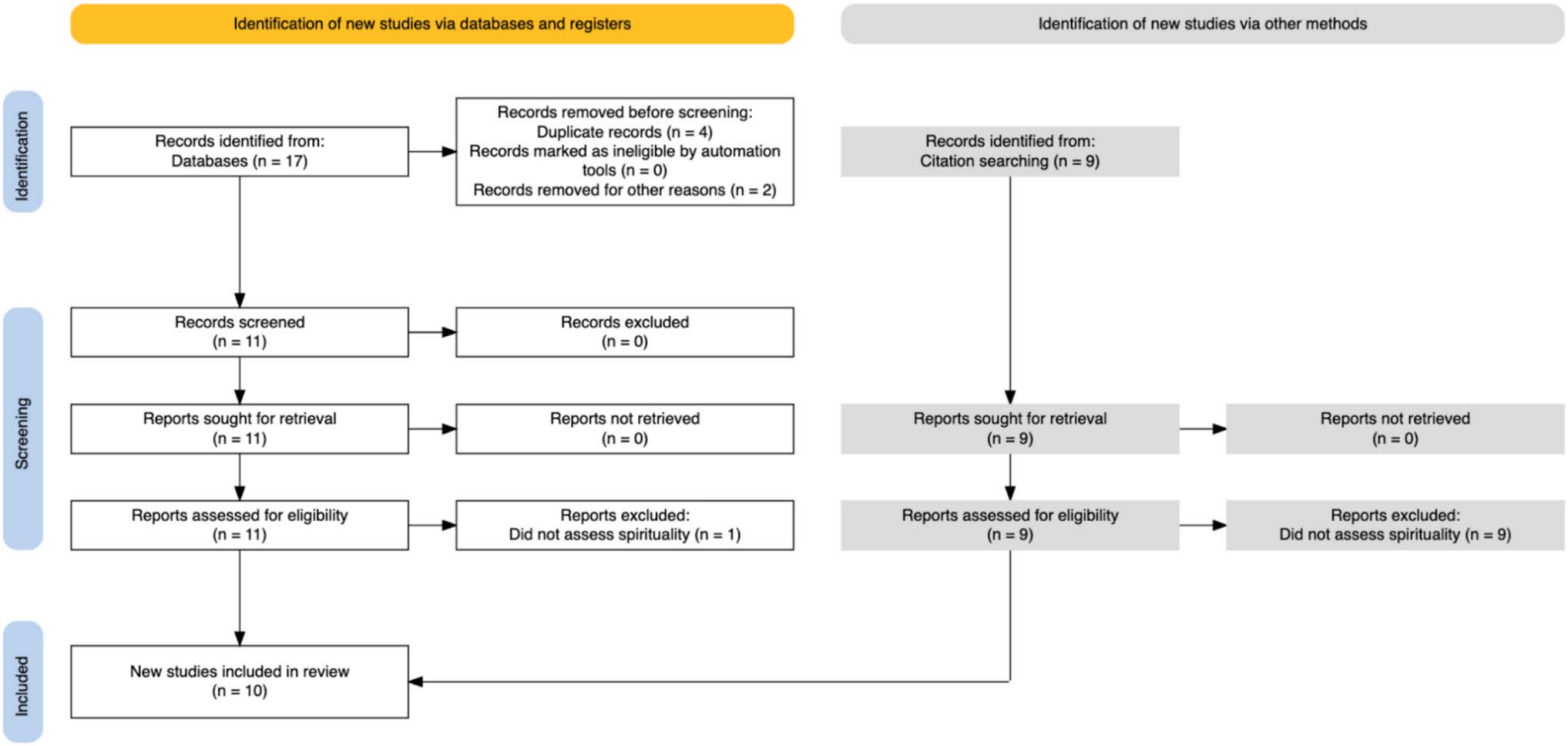
PRISMA flow chart showing the article selection process

This is a scoping review following the PRISMA Extension for Scoping Reviews (PRISMA-ScR) guidelines [11]. The search plan included a comprehensive electronic search of PubMed, Scopus, PsycINFO, Ovid MEDLINE and Cochrane Library with the words “Systemic Sclerosis” OR “Scleroderma” AND “spirituality” up to April of 2025. Gray literature (eg. conference proceedings, thesis, government reports, clinical trial data) were not included in this review. Two of the authors (SG, FP) did an initial independent search and each one identified a set of articles based on the search terms used and then screened by title and abstract to exclude publications which were duplicate or not relevant to the study. Three of the authors (SG, FP, CTD) came to a consensus of which articles to further screen and also which of the references of these articles also needed to go through further screening. Three of the authors (SG, FP, CTD) then did another independent full-text screen of both the selected articles as well as related references to exclude studies that did not assess or report on spirituality. The three authors (SG, FP, CTD) then came to a final consensus as to which articles to be included in the study. We did not specifically screen out articles written in languages other than English but the final set of articles which was selected for the scoping review were all in English. (Fig. 1).

Two of the authors (SG, FP) independently reviewed the 10 selected articles and extracted key information. Each researcher’s individual work was then reviewed by the other to ensure accuracy and consistency. A third author (CTD) then reviewed and finalized the accuracy and consistency of the data collected and in areas of disagreement a consensus of the three authors (SG,FP,CTD) prevailed.

### Study selection

Original, peer-reviewed, published studies were included that evaluated or reported on spirituality in SSc patients, using any available data collection instrument, to ensure that important and relevant findings are not omitted. No restrictions were placed on study design or setting.

The inclusion criteria were: 1) studies focused on spirituality or spiritual well-being in patients with a SSc diagnosis, 2) studies should stem from original research 3) studies were published in a peer-reviewed journal.

Exclusion criteria intentionally were limited as the bibliography on the subject is rather scarce. All studies relevant to the subject matter were included, in order to achieve a broad image on the subject and allow for transparency into the unmet spiritual needs of SSc patients.

Some included studies enrolled mixed cohorts (patients with systemic sclerosis, lupus, psoriasis, or melanoma). In our synthesis, we clearly distinguished findings specific to SSc from those that applied to broader populations, acknowledging this as a limitation. In addition, although our initial protocol excluded gray literature, we performed a complementary search in Google Scholar, which indexes non-peer-reviewed sources. This search did not yield additional relevant studies. However, we note that excluding gray literature may limit comprehensiveness and introduce potential publication bias.

### Data extraction and assessment of methodological quality of study

From the selected studies data were extracted on the year of publication, the self-administered instrument used, the country where the work was done, and the number of SSc patients who took the self-administered questionnaire. We documented how each study classified SSc patients, using either the 1980 American Rheumatism Association (ARA) criteria [12], the 2013 American College of Rheumatology/European League Against Rheumatism (ACR/EULAR) criteria [13], or other classification methods. For demographics, we collected data on the mean age of the study population, gender and SSc subtype. The Joanna Briggs Institute (JBI) instrument [14] was used to evaluate the methodological quality, design, and reporting of the selected studies. This tool consists of eleven key items: (1) clearly and explicitly stated review question, (2) inclusion criteria appropriate for the review question, (3) appropriate search strategy, (4) adequate sources and resources used to search for studies, (5) appropriate criteria for appraising studies, (6) critical appraisal conducted by two or more reviewers independently, (7) methods to minimize errors in data extraction, (8) appropriate methods to combine studies, (9) assessment of publication bias, (10) recommendations for policy or practice supported by the data, and (11) identification of appropriate directives for future research. Each item was scored as 1 (yes) or 0 (no, unclear, or not applicable). In Table 1, we report the total JBI score for each article used in this review, with a possible range from 0 to 11.

**Table 1:** Articles used for scoping review evaluating spirituality in SSc

Study	Population	Sample size	Demographics	Instrument	Objective	Key results	JBL level of evidence	Classification method
Quantitative studies								
Chen et al., 2023	USA patients with SSc (UCLA), secondary analysis of a previous Quality of Life study	206	84% female, 52 mean age, 74% Caucasian, 52% LcSSc, 41.8% DcSSc	FACIT-Sp	To examine how functional limitations, social support, and spiritual well-being relate to life satisfaction in people with systemic sclerosis, and whether social support or spiritual well-being moderate the impact of functional limitations on life satisfaction.	Spiritual well-being was the strongest positive predictor of life satisfaction among patients with SSc. While it did not moderate the relationship between functional limitations and life satisfaction, its direct association highlights its importance in overall well-being.	4	2013 ACR/EULAR
Iani et al., 2020	Italian Patients with SSc or Psoriasis (PsO)	192 (41 SSc)	97.8% female, mean age 56.5, (SSc patients)	FACIT-Sp	To examine how aspects of positive and negative functioning (e.g., reappraisal, sense of coherence (SOC), positivity, expressive suppression) relate to spiritual well-being and psychological distress in individuals with PsO and SSc.	Positivity was the strongest predictor of higher spiritual well-being, followed by subcomponents of SOC. High SOC and reappraisal were linked to better spiritual outcomes, while high skin symptoms and expressive suppression were linked to higher psychological distress. Results suggest the value of enhancing inner strengths to support spiritual well-being in skin disease patients.	4	Not defined
Unterrainer et al., 2016	Austrian patients with severe skin diseases (SSc/SLE/Melanoma)	149 (44 SSc)	84% female, 23–80 age range, 59% LcSSc, 16% DcSSc (SSc patients)	MI-RSWB	To examine spirituality and mood pathology in severe skin conditions including SSc.	The study found that patients with SSc had significantly higher levels of somatization and depression compared to those with SLE and malignant melanoma, indicating a greater psychiatric burden. Across all three patient groups, lower psychological symptomatology was associated with higher levels of spiritual well-being, particularly hope for a better future and belief in a better afterlife. These specific spiritual dimensions appeared to act as protective factors against mood disturbances.	2	Not defined
Pilch et al., 2016	Austrian patients with severe skin diseases (SSc/SLE/Melanoma)	149 (44 SSc)	84% female, 23–80 age range, 59% LcSSc, 16% DcSSc (SSc patients)	MI-RSWB	To examine coping strategies and the role of religiosity/spirituality in improving subjective well-being in SSc, SLE, and melanoma.	At diagnosis, patients with SLE experience a greater disease burden than those with SSc or melanoma, though SSc and SLE patients typically require several years to come to terms with their illness. SLE patients report significantly lower well-being, and their symptoms like photosensitivity and joint pain are negatively linked to forgiveness. SSc patients with facial or lung involvement tend to be more religious, while melanoma patients exhibit greater transcendental hope.	2	Not defined
Iwamoto et al., 2011	Japanese Patients with intractable diseases incl. SSc, SLE	44	50% female, 47.2 mean age	Self-Transcendence Scale (STS), WHO-SUBI	Assess self-transcendence and well-being	Higher self-transcendence is associated with better subjective well-being. Patients with intractable diseases had significantly higher scores in self-transcendence compared to the healthy controls. This indicates that chronic illnesses may stimulate spiritual reflection. Although the exact number of SSc patients in the sample was not mentioned, the study included a wide range of autoimmune diseases.	2	Not defined
Rubenzik et al, 2009	USA patients with SSc from Thomas Jefferson University	25	92 % female, 51.0 mean age, 84% Caucasian, 36% LcSSc, 36 % DcSSc	SScNQ (includes spiritual domains)	To evaluate the unmet needs of patients with systemic sclerosis from their own perspective using a questionnaire, and to identify which demographic factors are associated with these needs	The greatest prevalence of unmet needs in scleroderma patients were in the psychologic/spiritual/existential domain, such as being unable to do things they used to do, fear that the disease will worsen, anxiety and stress, feeling down or depressed, fears of physical disability, uncertainty about the future, change in appearance, keeping a positive outlook, and feeling in control.	2	Physician Evaluation
Provencher et al., 2023	USA and Canadian patients with SSc attending support groups	175	86% female, 56.4 mean age, 86% Caucasian, 57% LcSSc, 29% DcSSc,grouped by years since diagnosis	Custom survey (non-validated spirituality question)	To evaluate whether informational and support needs, including spirituality, differ among people with systemic sclerosis based on time since diagnosis.	Spirituality was considered a relevant support need, especially among those recently diagnosed (0–3 years). This group rated spirituality and other personal concerns (e.g., family, finances, sexual issues) as more important than those with longer-standing diagnoses. Overall, support needs were high across all durations.	11	Not defined
Qualitative studies								
Gholizadeh et al., 2018	USA patients with SSc from UCLA, UCSD, Virginia Mason	114	86.8% female, 49.5 mean age, 68.4% Caucasian,	Open-ended questions	To explore how patients with scleroderma perceive the causes of their illness through open-ended qualitative responses.	Patients attributed scleroderma to diverse causes, including stress, environment, genetics, and spirituality. Stress was the most commonly cited factor. The inclusion of spirituality as a perceived cause highlights the need to consider patients’ personal belief systems in disease adjustment and support.	2	2013 ACR/EULAR
Hornboonherm et al., 2017	Thai SSc patients at Khon Kaen University	12	66.7% female, 54.3 mean age	Interviews and observation	To study self-care behaviors and trajectory management in northeastern Thailand among SSc patients.	Thai patients with systemic sclerosis developed tailored self-care strategies aligned with three distinct illness trajectories: (1) long-term stability becoming unstable, (2) intermittently unstable phases, and (3) continuous instability with periodic stability. The patients created personalized self-care methods that fit with these trajectories, including cultural healing traditions and addressing existential issues, demonstrating the incorporation of spiritual elements into their self-care activities.	2	Physician Evaluation
Expert opinion studies								
Finlay et al., 2021	Working group of the European Academy of Dermatology and Venereaology (EADV) task force on Quality of Life and Patient Oriented Outcomes (QoL/PO).	8 Dermatologists, 1 Psychologist, 1 Epedimiologist, 1 Pharmaco-Epidemiologist	Not specified	Position Statement (Expert report)	Review non-drug interventions to improve QoL	The association between increased psychological burden and lower spiritual well-being was described by the group. Religious and spiritual coping mechanisms could potentially improve emotional well-being and quality of life in patients with chronic skin diseases including SSc.	3	Not defined

**Fig. 2:**
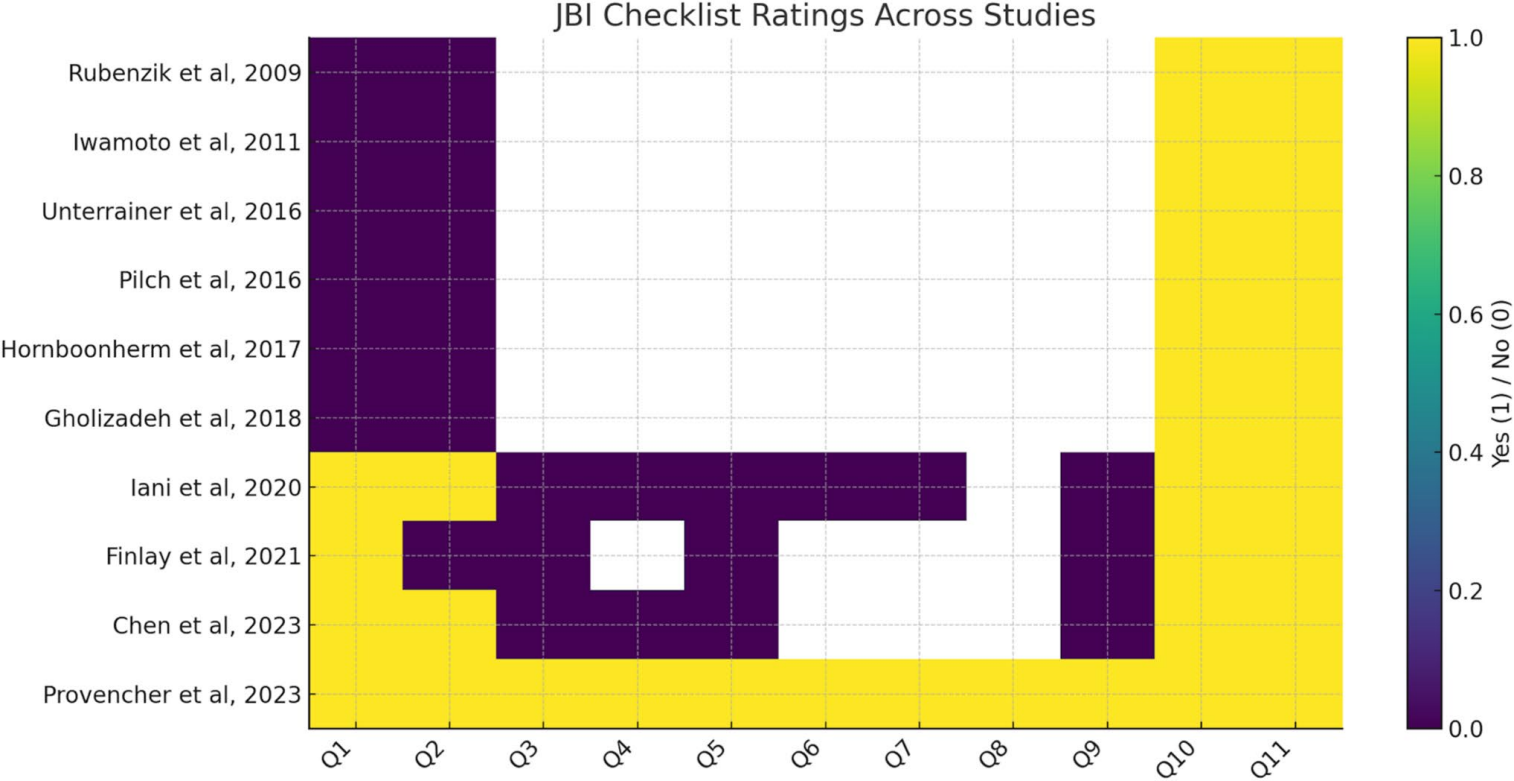
Heatmap of domain-level Joanna Briggs Institute (JBI) checklist ratings across included studies. Each study was assessed across 11 JBI domains (Q1–Q11), with “Yes” responses shown in yellow, “No” responses in purple, and “N/A/Unclear” in white. The visualization highlights methodological strengths and weaknesses of individual studies

Using the Joanna Briggs Institute (JBI) checklist, each study was rated across 11 individual domains. Across studies, common strengths included clearly stated review questions and appropriate inclusion criteria. However, methodological weaknesses were frequent: most studies were cross-sectional with small sample sizes, few described strategies to minimize bias in data extraction, and none provided an assessment of publication bias. Only a minority of studies explicitly described independent critical appraisal by two or more reviewers. Full ratings for each JBI domain are provided in Supplementary Table S2, with a graphical summary included in Fig. 2. Inter-rater reliability for JBI scoring was not formally calculated, which represents a limitation of our review process.

## Results

We identified seventeen records based on the above search methodology, of these four were duplicate and were removed while two did not relate to our inclusion criteria. The remaining eleven records were reviewed in full text, and one study was excluded as it did not assess spirituality. From the initial seventeen records another nine possible records were identified based on the citations. All nine of these records were dismissed after a full review on the grounds that they did not assess spirituality. Thus, the remaining ten records were reviewed for our study and summarized below and on Table 1.

In a 2009 study, Rubenzik et al., evaluated the unmet needs of SSc patients based on the patients’ perspective to better identify and characterize the demographic factors which are associated with these needs [15]. From a total of 242 actively followed SSc patients at a rheumatology clinic, fifty of them were randomly selected by a computer randomization program. An unmet needs survey was sent to all fifty patients, of whom 25 responded to the survey. The survey was modified by a validated SLE unmet needs questionnaire which consisted of 81 questions with 9 providing demographic data and 72 addressing various aspects including physical form, daily living, psychological and spiritual needs, existential matters, health information and services, social support and employment issues. The results revealed that the spiritual, existential and psychological category had the highest number of unmet needs, with overall 9 questions reaching significance. Fears of worsening disease, limitations on the activities they used to perform, anxiety, stress and depression as well as physical disabilities and uncertainty about the future were some of the categories examined. Changes in appearance, difficulty keeping a positive outlook, and feeling of lack of control were also evaluated. The health services category had 5 significant questions, whereas the physical category had 4. Patients who had not attended college were more likely to report higher needs than those with a college degree. Unmarried patients reported higher needs in 8 categories compared to married people, and patients in rural areas had higher needs in social support.

In a cross-sectional study in 2011, Iwamoto and colleagues explored the association of self-transcendence, which is conceptualized as the spiritual idea of the sense beyond the self and the subjective well-being in 44 patients with autoimmune diseases such as SSc and SLE [16]. The authors aimed to understand whether patients who experience ongoing physical suffering might develop higher levels of self-transcendence and whether it is related to their overall health. They assessed patients using the Self-Transcendence Scale (STS) and the World Health Organization Subjective Well-Being Inventory (WHO-SUBI), comparing results between patient and healthy control groups. The results suggested that patients with intractable diseases had significantly higher scores in self-transcendence compared to the healthy controls. This indicates that chronic illnesses may stimulate spiritual reflection. Additionally, higher self-transcendence was positively correlated with subjective well-being. Although the exact number of SSc patients in the sample was not specified, the study included a wide range of autoimmune diseases. The study highlights that supportive care practices in patients with chronic illness may foster self- transcendence and spiritual growth.

In the prospective observational study of 2016 by Unterrainer et al., the association between the psychological distress and spirituality of patients with severe skin disorders, such as SSc, SLE and malignant melanoma, was investigated [17]. The patients were recruited from the Department of Dermatology and Venereology at the medical University of Graz in Austria. The Multidimensional inventory for Religious/Spiritual Well-Being (MI-RSWB) was used to examine spiritual well-being and its subscales such as religiosity, hope and forgiveness, connectivity and the general feeling of meaningfulness. Psychological distress was assessed using the Brief Symptom Inventory (BSI), which evaluates a variety of psychiatric symptoms. Generally, strong negative connections between spiritual well-being and psychological discomfort have been suggested. Higher scores on the MI-RSWB subscales were associated with less psychiatric problems, indicating that spirituality might act as a protective factor against mood instability in people with skin disorders. The study found that patients with SSc exhibited significantly higher levels of somatization and depression compared to those with SLE and malignant melanoma, indicating a greater psychiatric burden. Across all three patient groups, reduced psychological symptomatology was associated with higher scores of spiritual well-being, particularly hope for a better future and belief in afterlife. These specific spiritual dimensions appeared to act as protective factors against mood disturbances.

Pilch et al. (2016) assessed the function of religious and spiritual well-being in relation to coping with chronic skin disorders like SSc, SLE and malignant melanoma [18]. (This study appears to be a secondary analysis of the same patient cohort described in Unterrainer et al.). The participants were requested to fill out a self-designed survey about personal well-being and settings surrounding their chronic disease, as well as the MI-RSWB. The main goal was to investigate the relationship between spiritual, religious well-being to patients’ adaptation to illness. At the time of the diagnosis, SLE patients were more heavily affected by the illness rather than their SSc counterparts. Acceptance of the disease diagnosis took a whole year after initial diagnosis in 72% of SSc and 74% of SLE patients, but most melanoma patients came to terms with it within 3 months. Generally, SLE patients had poorer total religiosity/spirituality (R-S) well-being ratings, while photosensitivity and joint pain were found to be inversely connected to forgiveness. SSc patients who suffered from pulmonary involvement and face lesions showed higher religiosity. Melanoma patients demonstrated higher transcendental hope scores. The authors concluded that patients suffering from either one of the three diseases require extended psychological support. Although structured programs focused on improving religious/spiritual coping skills are not widely available, the authors noted that such programs could be useful tools for improving patient well-being in the years to come after diagnosis.

In a 2017 qualitative study, Hornboonherm et al. investigated self-care behaviors and disease trajectory control among people suffering from SSc in Northeastern Thailand [19]. The study used an exploratory case study model with twelve selected patients from the specialized scleroderma clinic at Srinagarind Hospital, Faculty of Medicine, Khon Kaen University. Data was gathered through interviews, outpatient record reviews and overall observations, and were evaluated using content analysis. The analysis resulted into three different categories; 1. A continuous long-term stable phase transitioning into an unstable phase, characterized by mild and slowly progressing pathology: 2. A long-term stable phase with short interruptions of instability, involving a typical disease pattern without any changes in the visceral organs; 3. Cyclic changes between short unstable and stable phases, involving typical organ involvement and visceral organ disorders such as cardiac and pulmonary complications. The patients adapted their self-care methods according to these trajectories, incorporating cultural healing traditions and addressing existential concerns.

In a qualitative study conducted in 2018, Gholizadeh et al. investigated, through open-ended questions, the way people with SSc perceive the origins of their illness [20]. Content analysis was the method that the researchers used to identify and analyze the following categories in the patient’s illness causality: stress (36%), environment (27%), genetics (21%), medical problems or procedures (18%), food (7%), drugs or substance use (7%), and spirituality (3%). Stress surfaced as the most often reported trigger, with patients recalling a plethora of both acute and chronic causes, such as pressure in the family and work environment, personal psychological problems, like chronic anxiety. Exposure to chemicals or harsh weather conditions were included in the list of environmental contributors. A genetic profile of the diseases was, also, noticed by the patients, especially those with a family history of some autoimmune disease**.** Lastly, a small percentage of patients linked their existing condition to spiritual elements, implying that their condition was linked to spiritual trial or a divine purpose.

In the 2020 cross-sectional study of Iani et. al., it was examined whether aspects of positive functioning were associated to spiritual well-being and level of psychological distress in patients with Psoriasis and SSc [21]. Positivity, sense of coherence (SOC) and cognitive reappraisal were examined. Key constructs such as skin symptoms severity, the sense of coherence, the level of psychological distress, and spiritual well-being (as measured by the FACIT-Sp scale) as well as techniques to manage the underlying disease were documented. The predictive value of these variables on psychological distress and spiritual well-being was assessed using hierarchical multiple regression analysis. Maintaining a positive mindset was the strongest indicator of higher spiritual well-being, followed by the comprehensibility/manageability and meaningfulness components of SOC. In contrast, higher degrees of skin-related symptoms and expressive inhibition were linked to increased psychological discomfort**.** Although cognitive reappraisal was also associated with better psychological outcomes, it did not significantly predict spiritual well-being. Improving inner qualities, such as optimism might promote better spiritual well-being and lower the levels of psychological distress caused by such skin diseases.

In their 2021 position statement, Finlay and colleagues addressed the importance of non- pharmacological interventions in enhancing the quality of life for patients suffering from skin diseases, including SSc, SLE and melanoma [22]. The authors noted that besides medical treatment, spiritual well-being plays a crucial role in the overall patient health. Holistic approaches, such as spiritual assessments into patient care to address the multiple needs of individuals with skin conditions were deemed important. This position statement suggested the association between increased psychological burden and lower spiritual well-being. Religious and spiritual coping mechanisms could potentially improve emotional well-being and quality of life in these patients. Although specific demographic data were not detailed, the authors suggested further research should be performed to explore the impact of spiritual well-being on patient outcomes. They recommended that healthcare providers consider incorporating spiritual assessments into routine care.

The cross-sectional survey conducted by Provencher et al. in 2015 and published in 2023 investigated whether the support and informational needs of people with SSc vary based on the duration since diagnosis [23]. A 30-item questionnaire was completed by 175 SSc patients from the USA and Canada, focusing on reasons for attending support groups. After being divided into three groups, based on whether they got their diagnosis between 0–3 years, 4–9 years, and 10 or more years, the authors dichotomized the survey responses into "Not Important or Somewhat Important" versus "Important or Very Important," and statistical analyses were performed to identify differences.

The conclusion of the study was that regardless of the duration of the diagnosis, most support needs were deemed as "Important" or "Very Important," especially those related to social (median 81%) and interpersonal support, as well as learning more on the disease (median 82%), and strategies of management. On the other hand, topics such as spirituality, talking with family and friends, financial regards and sexual health were rated lower overall (median 44%). However, patients diagnosed within the past three years rated the above factors significantly higher than those diagnosed earlier, with statistically significant differences in the medical care area, spirituality, talking with family and friends, financial and sexual issues. The findings suggest that patients who are newly diagnosed with SSc have higher demands regarding the informational and support needs, a fact that emphasizes the importance of timely support interventions.

In the 2023 study by Chen et al., researchers evaluated the association between social support, functional limitations, spiritual well-being and life satisfaction in patients with SSc [24]. Using questionnaires, the participants assessed the demographics of the disease, depressive symptoms (using the Center of Epidemiologic Studies Depression Scale (CES-D 10)), functional limitations (using the Health Assessment Questionnaire Disability Index, (HAQ-DI)), social support (using the Medical Outcomes Study Social Support Survey (MOS-SSS)), spiritual well-being (using the Functional Assessment of Chronic Illness Therapy-Spiritual Well-Being Scale (FACIT-Sp)), and life satisfaction (using the Satisfaction with Life Scale (SWLS)). Hierarchical linear regression analyses were conducted to evaluate the links between these variables.

The conclusions of these reports found that 38% of participants reported being dissatisfied with their lives. Functional limitations were associated with a lower life satisfaction, while considerable social support and spiritual well-being had a positive effect on satisfaction in everyday life. Among these factors, spiritual well-being emerged as the strongest predictor. However, neither a strong social support system nor spiritual well-being moderated significantly the status of functional limitations. Non-married patients, racial minorities and those experiencing depressive symptoms had lower life satisfaction.

### Thematic synthesis of findings

The 10 studies included in this review highlight the role of spirituality in the lives of SSc patients as an existing and multifaceted one. The conduction of thematic analysis on the above-mentioned results revealed three primary themes: 1) spirituality as a coping mechanism 2) variability of spiritual expression, 3) neglect of spirituality in patient care.

Spirituality may represent a potentially meaningful component of holistic care in systemic sclerosis, but current findings should be considered hypothesis-generating given the limited and heterogeneous evidence base. Spirituality appears as a way out of strain and a relief from the uncertainty that comes with a chronic disease.

A higher state of spirituality, the one that encompasses hope, meaning, transcendence, is linked to lower percentages of depression and somatization of it among the SSc patients [15, 17, 21, 24].

The meaning of spirituality though it can theoretically be defined, takes different forms and degrees of severity depending on demographics such as cultural and educational background, social support and time since diagnosis [18–20, 23].

Even though the value of spirituality has become apparent, it remains marginalized in the clinical care of patients with SSc. [15, 22, 24] Each one of the studies reviewed calls for further integration of spirituality in patient care.

On domain-level appraisal using the Joanna Briggs Institute (JBI) checklist, most studies were cross-sectional with small sample sizes and limited stratification of SSc patients. Common strengths included clearly stated research questions and appropriate inclusion criteria. However, weaknesses were frequent: few studies described strategies to minimize bias in data extraction, none provided an assessment of publication bias, and only a minority explicitly described independent critical appraisal by two or more reviewers. A qualitative summary of risk of bias across studies therefore indicates modest methodological rigor overall. Full domain-level ratings are provided in Supplementary Table S2, with a graphical summary in Fig. 2.

## Discussion

Spiritual well-being is increasingly recognized as a critical domain of health in patients with chronic illnesses, including systemic sclerosis (SSc). As a concept, it encompasses how individuals find meaning, purpose, peace, and connection during illness and suffering. This scoping review synthesizes findings from ten published studies and aims to highlight the emerging but still underexplored role of spirituality in patients living with SSc.

Through the included studies, spiritual well-being was consistently associated with psychological resilience and improved quality of life. Spiritual well-being, as measured by the FACIT-Sp, was a potential contributor to life satisfaction in some studies (Chen et al. 2023), though findings remain preliminary given small, heterogenous samples [25]. Higher religious/spiritual well-being was inversely related to depression, anxiety, and somatization symptoms [17], and it was observed that patients with chronic illnesses, including SSc, had high levels of self-transcendence, which were positively associated with well-being [16]. In a similar vein, qualitative interviews in Thai SSc patients, revealed that spirituality was closely linked with self-care and acceptance of illness, often grounded in Buddhist cultural values [19]. These findings underscore that the expression and role of spirituality vary across cultural and religious contexts, influencing how patients conceptualize coping and meaning-making. For example, spirituality in Buddhist contexts often integrates with self-care and acceptance, whereas in Western cohorts spirituality may be framed through religious faith or existential reflection. Future studies should critically examine these cultural dimensions rather than treating spirituality as a uniform construct.

Other studies contributed unique insights into how patients with SSc conceptualize and integrate spirituality. Some patients interpreted their disease as a spiritual test or punishment, which shaped both their emotional coping and care preferences [21]. In contrast, in another study, spirituality was ranked by patients as the most helpful coping strategy among a list of common supports, such as family and medications [15]. Another study highlighted the complexity of spiritual distress, noting that patients with severe dermatologic disease (including SSc) experienced spiritual struggles that were not always addressed in care [18]. At the same time, patients with chronic autoimmune diseases, including SSc, exhibited high dispositional optimism and purpose in life, supporting the idea that spirituality may be intertwined with psychological resilience [22]. Provencher et al. demonstrated that spiritual well-being remained relatively stable regardless of disease duration, suggesting it may be an enduring resource [23]. Finally, other researchers emphasized the importance of integrating spirituality into care models and medical education, calling for formal spiritual screening in rheumatologic settings [20].

Several validated instruments were used across studies to assess spirituality, each offering different conceptual emphases. The most utilized tool was the Functional Assessment of Chronic Illness Therapy–Spiritual Well-Being Scale (FACIT-Sp), which evaluates three domains: meaning, peace, and faith. It has been widely validated in chronic illness populations and demonstrates strong psychometric properties [25]. The Multidimensional Inventory for Religious/Spiritual Well-Being (MI-RSWB) was also used, offering broader assessment across hope, forgiveness, and existential connectedness [26]. Iwamoto et al. employed the Self-Transcendence Scale (STS), which captures a sense of meaning and purpose beyond the self [27]. Other tools, such as the World Health Organization Subjective Well-Being Inventory (WHO-SUBI) and Short Form Health Survey (SF-36), provided complementary insight into emotional or existential health but were not specifically designed for spirituality [26, 28]. The use of diverse instruments highlights the growing interest in measuring spirituality but also underscores the need for consistent, validated, and culturally adaptable tools in future research.

Taken together, the findings suggest that spirituality is a relevant and influential factor in the lived experience of SSc. It affects not only how patients understand and cope with their illness but also how they engage with healthcare systems. Integrating spirituality into established psychosocial frameworks — such as resilience, coping strategies, and patient-centered care — may help situate these findings within a broader understanding of chronic illness adaptation. Given the chronic, unpredictable, and often disfiguring nature of SSc, clinicians should consider routinely assessing spiritual well-being and offering resources such as chaplaincy referrals, support groups, or spiritually integrated psychotherapy when appropriate. Future research should aim to standardize assessment tools in order to measure spiritual well-being, understanding how cultural and religious backgrounds shape patients. Furthermore, test whether integrating spiritual support in the care of rheumatological patients improves health outcomes, explore the influence of cultural, religious context, and rigorously evaluate interventions that integrate spirituality into comprehensive rheumatologic care.

### Strengths and limitations

This review offers a variety of strengths in methodology. It was based on the PRISMA-ScR guidelines and a immersive research in major databases was held, ensuring a solid capture of relevant literature. It includes both quantitative and qualitative research, leading to a holistic and multidimensional understanding on the delicate subject of spirituality among SSc patients. Furthermore, the use of JBI tool contributes to the assessment of studies’ quality. What is more, the intentional decision to include all available bibliography, even the publications that shortly addressed spirituality, allows for identification of key gaps in this area of interest.

Nonetheless, limitations should also be acknowledged and enumerated. The number of studies available was short, probably limiting its ability to include all SSc patients. Moreover, not all studies primarily focus on spirituality but rather address it as a secondary matter, leading to varying depth of the results. Finally, the implementation of spiritual care into clinical practice is yet to be tested, so any suggestion remains strictly on a theoretical background.

It is important to note that several included studies recruited mixed patient populations (e.g., SSc, lupus, melanoma, or other chronic conditions). Where possible, SSc-specific findings were described separately; however, in studies without stratified results, conclusions must be interpreted cautiously and findings should be considered hypothesis-generating.

We also excluded gray literature, although a targeted Google Scholar search was performed to identify potential theses, conference abstracts, or reports. No additional relevant sources were identified. Nevertheless, this exclusion remains a limitation and may have introduced publication bias. In addition, while non-English studies were not excluded a priori, all included works were published in English. This may reflect limitations in indexing or search strategy and may bias the evidence base toward Western populations.

This review is limited by the small sample sizes of included studies, which reduce statistical power and generalizability. Considerable heterogeneity exists in study populations, instruments used to measure spirituality, and analytic approaches, complicating cross-study comparisons. In addition, the exclusion of gray literature and the small number of available studies raise the risk of publication bias, whereby positive findings are more likely to be reported. These factors collectively suggest that the present results should be considered hypothesis-generating rather than definitive.

### Future steps

In order for these findings to be incorporated into clinical practice, several steps might be recommended. Clinical doctors could consider a brief open-ended conversation with their patients, regarding not only their standard treatment but also about meaning, mental strength and resilience. When needed, a chaplaincy service or psychotherapy could work alongside the primary caregivers, soothing the complexity of living with SSc. Finally, basic staff training on spiritual needs and preferences will hopefully create a healthcare environment that acknowledges spirituality as a fundament of holistic care.

## Supplementary Information

Below is the link to the electronic supplementary material.

Supplementary file1 (DOCX 56.5 KB)

Supplementary file2 (DOCX 17.3 KB)
