# Supplementary material for: Understanding spiritual well-being in patients with systemic sclerosis and its effect on the illness: a scoping review
Source: Clin Rheumatol. 2025 Sep 17;44(11):4553–66. doi: 10.1007/s10067-025-07689-1 (PMC12568786; doi:10.1007/s10067-025-07689-1)
Supplement: Supplementary file 2 — Supplementary file2 (DOCX 17.3 KB) [file 10067_2025_7689_MOESM2_ESM.docx]

| Study (Author, Year) | Q1: Review question clearly stated | Q2: Inclusion criteria appropriate | Q3: Search strategy adequate | Q4: Sources/resources adequate | Q5: Appraisal criteria appropriate | Q6: Critical appraisal by > 1 reviewer | Q7: Methods to minimize extraction errors | Q8: Appropriate synthesis methods | Q9: Publication bias assessed | Q10: Recommendations supported by data | Q11: Future research identified |
| --- | --- | --- | --- | --- | --- | --- | --- | --- | --- | --- | --- |
| Rubenzik et al, 2009 | No | No | N/A | N/A | N/A | N/A | N/A | N/A | N/A | Yes | Yes |
| Iwamoto et al, 2011 | No | No | N/A | N/A | N/A | N/A | N/A | N/A | N/A | Yes | Yes |
| Unterrainer et al, 2016 | No | No | N/A | N/A | N/A | N/A | N/A | N/A | N/A | Yes | Yes |
| Pilch et al, 2016 | No | No | N/A | N/A | N/A | N/A | N/A | N/A | N/A | Yes | Yes |
| Hornboonherm et al, 2017 | No | No | N/A | N/A | N/A | N/A | N/A | N/A | N/A | Yes | Yes |
| Gholizadeh et al 2018 | No | No | N/A | N/A | N/A | N/A | N/A | N/A | N/A | Yes | Yes |
| Iani et al, 2020 | Yes | Yes | No | No | No | No | No | N/A | No | Yes | Yes |
| Finlay et al, 2021 | Yes | No | No | Unclear | No | N/A | N/A | N/A | No | Yes | Yes |
| Chen et al, 2023 | Yes | Yes | No | No | No | No | Unclear | N/A | No | Yes | Yes |
| Provencher et al, 2023 | Yes | Yes | Yes | Yes | Yes | Yes | Yes | Yes | Yes | Yes | Yes |

**Supplementary Table 2:** Domain-level Joanna Briggs Institute (JBI) checklist ratings for methodological quality of included studies. Each study was assessed across 11 JBI domains (Yes = 1, No/Unclear = 0). The table provides transparency into individual strengths and weaknesses rather than aggregate scores alone.
